# Genome-Wide Analysis of the NAC Domain Transcription Factor Gene Family in *Theobroma cacao*

**DOI:** 10.3390/genes11010035

**Published:** 2019-12-28

**Authors:** Shiya Shen, Qianru Zhang, Yu Shi, Zhenmei Sun, Qianqian Zhang, Sijia Hou, Rongling Wu, Libo Jiang, Xiyang Zhao, Yunqian Guo

**Affiliations:** 1 Center for Computational Biology, College of Biological Sciences and Technology, Beijing Forestry University, Beijing 100083, China; 2 State Key Laboratory of Tree Genetics and Breeding, Northeast Forestry University, Harbin 150000, China

**Keywords:** *Theobroma cacao*, Arabidopsis thaliana, NAC transcription factors, genome-wide analysis, bioinformatics

## Abstract

As a plant-specific transcription factor, the NAC (NAM, ATAF1/2 and CUC2) domain protein plays an important role in plant growth and development, as well as stress resistance. Based on the genomic data of the cacao tree, this study identified 102 cacao *NAC* genes and named them according to their location within the genome. The phylogeny of the protein sequence of the cacao tree *NAC* family was analyzed using various bioinformatic methods, and then divided into 12 subfamilies. Then, the amino-acid composition, physicochemical properties, genomic location, gene structure, conserved domains, and promoter cis-acting elements were analyzed. This study provides information on the evolution of the *TcNAC* gene and its possible functions, laying the foundation for further research on the *NAC* family.

## 1. Introduction

Transcription factors are proteins that control the rate of transcription of genetic information from DNA to messenger RNA, by binding to cis-acting promoter elements [1]. Each transcription factor contains at least one DNA-binding domain, which determines its main function in the gene expression regulatory network. According to their DNA binding domain, transcription factors in plants are divided into several families, such as *WKRY*, *bZIP*, *MYB*, *DREB*, *AP2/EREBP*, *C2H2*, *NAC*, etc. [2]. All of these transcription factors are essential for growth and development processes such as organ formation, secondary metabolism, hormone signaling, and the response to environmental stress [3,4].

The NAC domain protein is a plant-specific transcription factor first discovered in petunia [5]. The NAC domain protein is composed of an N-terminal DNA-binding domain, a nuclear localization signal sequence, and a C-terminal transcriptional activation domain. The N-terminal region is a conserved DNA domain, which comprises nearly 160 amino acids (aa) and can be further divided into five subdomains (A–E). The conservation of these five subdomains is in the order A > C > D > B > E, and subdomains A and C play a role in stabilization of the protein. The C-terminal region is a highly variable transcriptional regulatory region that interacts with DNA or other transcription factors [6,7]. NAC transcription factors have multiple functions in plants, such as the formation of plant shoot apical meristems [8,9,10], nutrient transfer [11], control of the cell cycle in plant senescence [11,12,13,14,15,16,17], the plant stress response [18,19,20], regulation of plant disease resistance and secondary growth [21,22], and hormone signaling [23,24]. In total, 166 species with *NAC* genes were identified. For example, there are 105 in *Arabidopsis* [25], 151 in *Oryza sativa* [26], 142 in *Vitis vinifera* [27], 163 in *Populus trichocarpa* [28], 113 in *Prunus mume* [29], 63 in *Coffea canephora* [30], and 152 in the soybean genome [31]. However, *NAC* genes were not studied in *Theobroma cacao*.

*Theobroma cacao*, also known as the cacao tree, belongs to the *Malvaceae* family and is one of the world′s three major beverage crops. Cocoa beans are the seeds of cacao trees and are the main raw material for chocolate. Globally, 3.7 million tons of cocoa beans are produced per year; however, diseases and pests cause harvest losses of about 30%. Determining the genes associated with cacao tree resistance is a key issue in its genetic breeding. Cacao trees also have high economic value because the cacao flavanols provide health benefits, which can be used in nutritional products [32,33,34], and cocoa polyphenols such as catechin and epicatechin have significant antioxidant properties and free radical-scavenging ability. To date, most studies of cacao trees focused on their active components; there were few studies at the genome level. The genome of the cacao tree was completed and published in 2011 [35], providing a powerful tool for studying the cacao tree at the gene level.

Many studies showed that, after plant stress, the NAC transcription factor family is involved in the regulation of responses to environmental stress [36]. Overexpression of *TsNAC1* in *Thellungiella salsuginea* can increase abiotic stress resistance [37], especially salt stress resistance. Tomato *JUN-GBRUNNEN1* directly binds to the promoters of *SlDREB1*, *SlDREB2*, and *SlDELLA*, increasing the drought tolerance of tomato [38]. *PtrNAC72* is a repressor of putrescine biosynthesis in *Poncirus trifoliata* and may negatively regulate drought stress responses by regulating putrescine-related reactive oxygen homeostasis [39]. Since the *NAC* gene family plays a crucial role in many developmental processes and responses to abiotic stresses, it is of great significance to study the *NAC* gene family in *Theobroma cacao*. In this study, we analyze the genetic structure, conserved motifs, chromosome localization, subcellular localization, and phylogenetic relationships of the *NAC* family members based on the annotation of *Theobroma cacao*. The results may be helpful for follow-up studies of the functional characteristics of the *NAC* gene family in *Theobroma cacao*.

## 2. Materials and Methods

### 2.1. Identification of *NAC* Family Genes in Theobroma cacao

*Theobroma cacao* genome sequences were downloaded from Ensembl Plants (http://plants.ensembl.org/index.html). The hidden Markov model (HMM) profile of the NAC domain (PF02365) was downloaded from the Pfam website (http://pfam.xfam.org/) [40]. We used the NAC HMM profile as the query to search against the *Theobroma cacao* genome sequence data. All protein sequences containing the NAC conserved domain were searched. To avoid missing *NAC* family members, we constructed a new HMM for *Theobroma cacao* using a high-quality protein set (E value < 1 × 10^−20^) for multiple sequence alignments in Clustal (Clustal 2.1; www.ebi.ac.uk). According to the aligned sequences, we constructed a new HMM in HMMER (HMMER 3.1; http://hmmer.org/) and used it as the query (E value < 0.01) to search against the *Theobroma cacao* genome sequence data. Genes encoding proteins with NAC domains were identified as *NAC* gene candidates. The ExPASy online program (http://web.expasy.org/translate/) was used to analyze the physicochemical properties of *TcNAC* genes. The BUSCA online program (http://busca.biocomp.unibo.it/) was used for predicting subcellular localization of proteins.

### 2.2. Sequence Analysis and Structural Characterization

Bioinformatic analysis of *TcNAC* gene sequences and the calculation of coding sequence (CDS) length, molecular weight (MW), isoelectric point (pI), and open reading frame (ORF) lengths was performed using the Compute pI/MW tool at the Expert Protein Analysis System (ExPAsy) site (http://au.expas y.org/tools/pi_tool.html). We used the GSDS (Gene Structure Display Server) tool to analyze the exon/intron organization for individual *NAC* genes of *Theobroma cacao* [41,42]. MEME (v.12.0; http://meme-suite.org/tools/meme) was used to analyze the motifs of TcNAC proteins with the following parameters: minimum width of motifs, six; maximum width of motifs, 50; and number of motifs, 10. TBtools (TBtools_v0.53.jar) was used to visualize the results.

### 2.3. Phylogenetic Analysis and Classification of the *TcNAC* Gene Family

MEGA 7.0 (http://www.megasoftware.net/) was used for constructing an individual phylogenetic tree of the *TcNAC* gene family [43]. Based on their aggregation with the *AtNAC* genes, the *TcNAC* genes were divided into different subgroups, and a comprehensive phylogenetic tree including *Arabidopsis* and *Theobroma cacao* was constructed using MEGA 7.0. All of the sequences were firstly aligned using ClustalW (http://www.ebi.ac.uk/clustalw/) with the default parameters [44]. Since not all *NAC* family members are necessarily homologous, to increase the reliability of the analysis, we deleted gaps and built a more conservative phylogenetic tree. Both of the phylogenetic trees were built using MEGA 7.0 using the maximum parsimony (MP) method [45] with 1000 repetitions for the bootstrap test [46].

### 2.4. Analysis of Cis-Acting Elements in the *TcNAC* Gene Promoter

Analysis of the cis-acting element in the *TcNAC* gene promoter was performed using TBtools software (v0.6669; http://cj-chen.github.io/tbtools/), which retrieved the upstream sequence (2.0 kb) of the *TcNAC* gene CDS from the cacao tree genome sequence, and converted it into the FASTA file format. The sequence was then submitted to PlantCARE (http://bioinformatics.psb.ugent.be/webtools/plantcare) in batches. The results were filtered to retain the response-related cis-acting elements, including the light-responsive, auxin-responsive, wound-responsive, and gibberellin-responsive elements (and the four corresponding originals). The results were visualized using TBtools.

### 2.5. Chromosomal Location and Evolutionary Analysis of *TcNAC* Genes

The DNA sequences of the *TcNAC* genes were downloaded from Ensembl Plants. Bio-Linux (v8.0.7; http://nebc.nerc.ac.uk/tools/bio-linux) was used for extracting information on chromosome length. MG2C (v2.1; http://mg2c.iask.in/mg2c_v2.1/) was used for the mapping the *TcNAC* genes according to their relative distances and chromosomal positions. The criteria for *TcNAC* gene duplication were as follows: (a) alignable sequence length >75% that of the longer genes, and (b) similarity of the aligned region >75% [47,48]. Clustal 2.1 was used for multiple alignments, and the KaKs calculator (ver. 2.0; http://code.google.com/p/kaks-calculator/wiki/KaKs_Calculator) was used for calculating Ka/Ks ratios.

## 3. Results

### 3.1. Identification of *TcNAC* Genes in Theobroma cacao

The *NAC* gene family is a transcription factor family unique to plants. NAC transcription factor sequences in *Theobroma cacao* were retrieved from the *Theobroma cacao* genome according to the HMM profile (PF02365) of the *NAC* family. Initially, a total of 136 non-redundant putative *NAC* genes were identified. After removing the redundant forms of the gene, a total of 102 genes were identified by HMM analysis (Supplementary File 1). Based on their chromosomal locations, the *TcNAC* genes were named sequentially from *TcNAC001* to *TcNAC102*. The physicochemical properties of the *TcNAC* genes were analyzed using the ExPASy online program. Detailed information on the *NAC* family genes in the cacao tree, including name and identifier (ID), number of aa, pIs, MW, ORF, and subcellular localization is provided in Supplementary File 2. The aa composition and physicochemical properties differ among the NAC family proteins, and the number of aa constituting proteins varies greatly among the different subfamilies. The protein sequences of the *TcNAC* genes were between 104 (*TcNAC018*) and 909 (*TcNAC013*) aa, with an average length of 341 aa. The predicted MW was between 15,933.7 Da and 103,748.5 Da, with an average MW of 38699.7 Da. The pI was between 4.14 (*TcNAC058*) and 9.97 (*TcNAC073*), with an average pI of 6.5. Overall, 64% of the TcNAC family proteins had a pI of less than 7. Therefore, the pI is in the acidic range and the proteins are rich in acidic aa. According to the results of the online software BUSCA, all the subcellular localizations of the cocoa tree NAC transcription factors could be predicted. Among them, 91 localized in the nucleus, five localized in the endomembrane system, and very few localized on the chloroplast (*TcNAC017*, *TcNAC101*), extracellular space (*TcNAC081*, *TcNAC096*), plasma membrane (*TcNAC088*), and mitochondrion (*TcNAC073*).

### 3.2. Phylogenetic Analysis and Classification of the NAC Gene Family in *Theobroma cacao* and *Arabidopsis*

**Figure 1:**
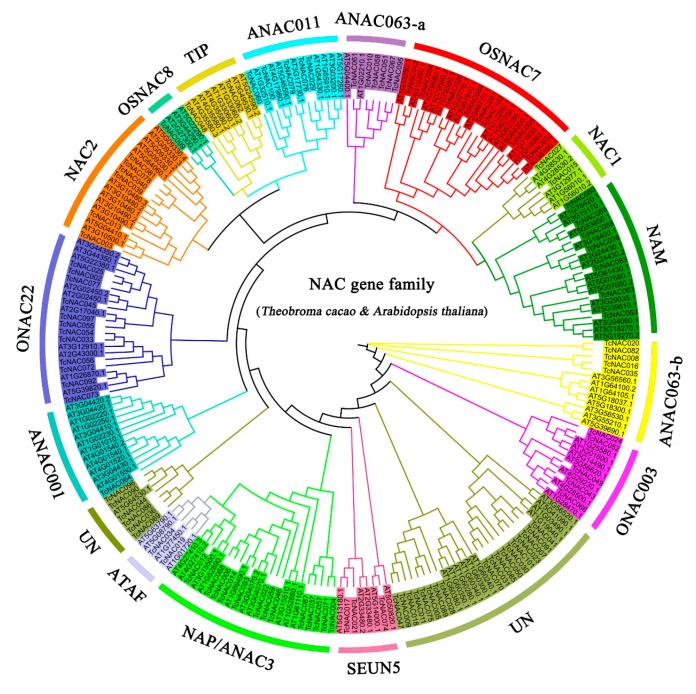
Phylogenetic tree of NAC (NAM, ATAF1/2 and CUC2) domain protein from *Arabidopsis* and *Theobroma cacao*. The phylogenetic tree was constructed using the maximum parsimony (MP) method with 1000 bootstrap replications. The 16 subfamilies are distinguished in different colors, and the unclassified TcNACs are represented by the abbreviation “UN”.

To examine the *NAC* gene family in *Arabidopsis*, rice, and cacao trees in evolutionary terms, and to analyze the characteristics of the cacao tree NAC protein, we used ClustalW to compare the aa sequences of 102 cocoa NAC proteins with 80 *Arabidopsis* NAC proteins. The phylogenetic trees were constructed using MEGA 7.0 by the neighbor joining, minimal evolution, and MP methods. The three phylogenetic trees obtained by the different methods were nearly identical. In this paper, the phylogenetic tree constructed using the MP method is presented. It was found that the *TcNAC* and *AtNAC* genes could be aggregated together, indicating that the *NAC* genes in the cacao tree that can be clustered with the *Arabidopsis* subgroup sequences are in the same subgroup (Figure 1). According to their homology with NAC proteins in *Arabidopsis*, the *NAC* family of cacao trees can be classified into 14 subfamilies: *NAM*, *NAC1*, *OsNAC7*, *ANAC011*, *TIP*, *OsNAC8*, *NAC2*, *ONAC022*, *ANAC001*, *ATAF*, *NAP/ANAC3*, *SEUN5*, *ONAC003*, and *ANAC063*. Moreover, those subfamilies are also conserved in rice [25] and *Tartary buckwheat* (*Fagopyrum tataricum*) [31]. Among these subfamilies, the *NAP/ANAC3* subfamily had the largest number of members, with 13 TcNAC proteins. The *OsNAC8* and *ANAC001* subfamilies had the fewest members, with only one TcNAC protein, and no members of the *ONAC001*, *TERN*, *ONAC002*, *AtNAC3*, and *OsNAC3* subfamilies were found in the cacao tree NAC protein.

Based on the phylogenetic tree, we hypothesized that the cacao tree NAC proteins in a subfamily with a common maternal lineage may have similar functions. For example, the *NAM* subfamily participates in the formation and development of shoot apical meristem [5,49], while *NAC* family members in the *ATAF*, *NAP*, *AtNAC3*, and *OsNAC3* subgroups have a conserved role in stress responses [50]. It is worth noting that there is only one branch of the *ANAC063* subfamily in *Arabidopsis*, which is divided into two parts (*ANAC063-a*, *ANAC063-b*) that are far apart in the phylogenetic tree. The TcNAC protein family also has two branches containing only the TcNAC protein, which has a distant relationship with the *Arabidopsis* protein within the same subfamily. This indicates that these genes have a long evolutionary relationship and differentiated long ago; the corresponding gene sequences since underwent many changes. During the development process, the cacao tree may gradually lose these genes, which may be related to the cocoa tree being a perennial woody plant. The phylogenetic tree derived in this study is consistent with *Arabidopsis*, indicating diverse functions of the *TcNAC* gene in the cacao tree.

### 3.3. *TcNAC* Gene Structure and Conserved Motifs

To study the structure of *TcNAC* genes, we analyzed their DNA sequences, and determined the composition of their introns and exons. The GSDS 2.0 software package (http://gsds.cbi.pku.edu.cn/) was used to map the intron–exon structure of the cacao tree *NAC* gene family. The results showed that 13 of the 102 *TcNAC* genes (9.8%) contained no introns. Among them, subfamily IV was shown to contain two genes without introns (accounting for 50% of the total number of genes in the subfamily); subfamily VIII contained one intron (20%), subfamily X contained seven introns (36.8%), and subfamily XI contained three introns (30%). Twelve of the *TcNAC* genes (11.8% of total genes) had multiple introns (range: 5–13; *TcNAC013* contained the largest number of introns, 13 introns). The gene structure analysis showed that gene length differed significantly among the cacao tree *NAC* gene family members. The shortest *TcNAC* gene was only 437 bp in length (*TcNAC096*), while the longest gene, *TcNAC013*, had a length of 7,831 bp. The gene structure of the *NAC* gene family was found to be moderately conserved among the various subfamilies, and the number and location of exons were similar among the *TcNAC* genes in each subfamily, indicating similar function. Most of the *TcNAC* genes had three introns.

**Figure 2:**
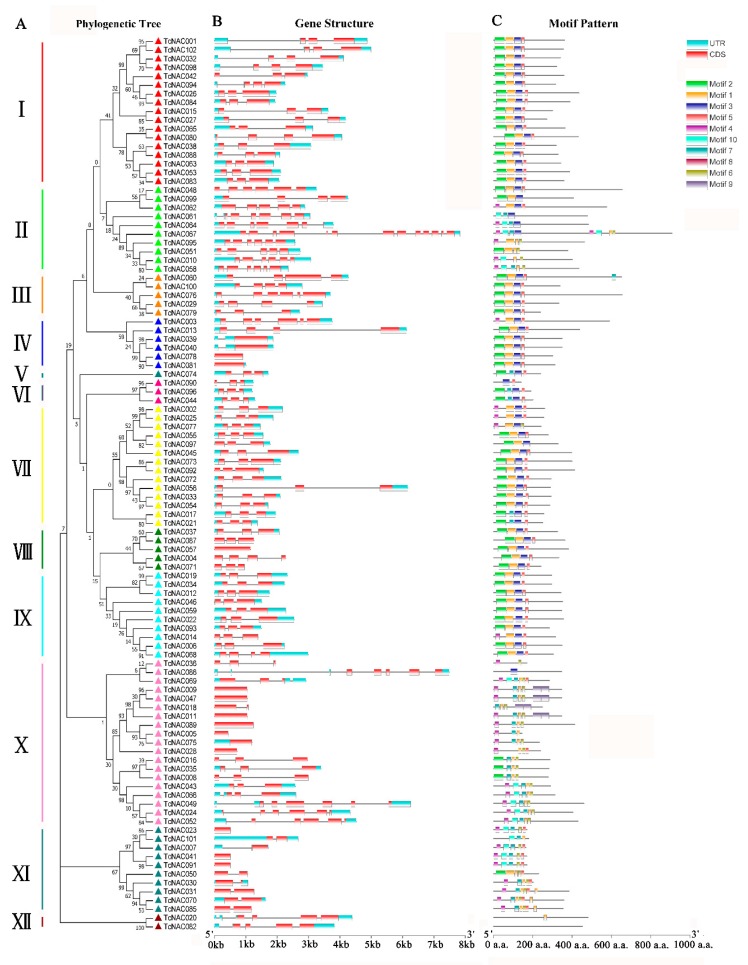
Phylogenetic relationships, gene structure and architecture of conserved protein motifs in *NAC* genes from *Theobroma cacao*. (**A**) The phylogenetic tree was constructed based on the full-length sequences of *Theobroma cacao* NAC proteins using MEGA 7.0 software. (**B**) Exon–intron structure of *Theobroma cacao NAC* genes. Blue boxes indicate untranslated 5′- and 3′-regions, red boxes indicate exons, and black lines indicate introns. (**C**) The motif composition of *Theobroma cacao* NAC proteins. The motifs are displayed in different colored boxes. The sequence information for each motif is provided in Supplementary File 3. The length of the protein can be estimated using the scale at the bottom.

To further study the diversity of TcNAC protein structure in *Theobroma cacao*, 10 conserved motifs (motifs 1–10) of the *Theobroma cacao NAC* family were identified using the Multiple Expectation Maximization for Motif Elicitation online program (MEME; http://meme.sdsc.edu/meme/meme.html). As shown in Figure 2, each member of subfamilies I and VIII contained the same motif type, and one gene each in subfamilies III, IV, VI, and IX was different. *TcNAC060* in subfamily III contained motif 7. *TcNAC003* in subfamily IV lacked motif 2, but contained motif 4. *TcNAC090* in subfamily VI lacked motifs 2 and 7. *TcNAC014* in subfamily IX lacked motif 2 but contained motif 4. The rest of the TcNAC proteins were identical. The results indicate that members of the *NAC* family belonging to the same subfamily have very similar motif types and numbers, but there are also differences in motif patterns among members of the same subfamily. Among the cacao tree NAC proteins, *TcNAC082* contained no motifs. *TCNAC086* and *TcNAC020* contained only one motif each (motifs 3 and 8, respectively). *TcNAC067* was found to be the most complicated protein, having eight motifs. Some motifs were sub-family specific, such as motif 9, which was only present in subfamily X. The finding of similar gene structures and conserved motifs within the same subfamily further supports the accuracy of the phylogenetic tree. On the other hand, the structural differences between different subfamilies also indicate functional diversity of the *NAC* gene family in cacao trees.

### 3.4. Responsive Elements in *TcNAC* Promoters

To further investigate the potential regulatory mechanisms of *TcNAC* during the abiotic stress response, a sequence 2 kb upstream from the translation initiation site of the *TcNAC* gene was submitted to PlantCARE to detect cis-elements. A total of 14 genes (*TcNAC010*, *TcNAC012*, *TcNAC019*, *TcNAC020*, *TcNAC030*, *TcNAC032*, *TcNAC050*, *TcNAC051*, *TcNAC054*, *TcNAC063*, *TcNAC082*, *TcNAC084*, *TcNAC086*, and *TcNAC095*) had no response elements, which proves that these genes may not be related to abiotic stress in the cocoa tree. Four response elements, a light-responsive element, an auxin-responsive element, a wound-responsive element, and a gibberellin-responsive element, were analyzed, and the results are shown in Supplementary File 4. *TcNAC001* and *TcNAC058* contained all four response elements, while the *TcNAC093* gene contained eight response elements, which was the largest number of response elements contained within one gene. There were 19 *TcNAC* genes that contained only one response element, accounting for 21.6% of all genes. Among the four response elements, the least prevalent was the wound-responsive element; only *TcNAC001*, *TcNAC058*, *TcNAC080*, and *TcNAC088* contained this element.

### 3.5. Chromosomal Location of *Theobroma cacao NAC* genes

**Figure 3:**
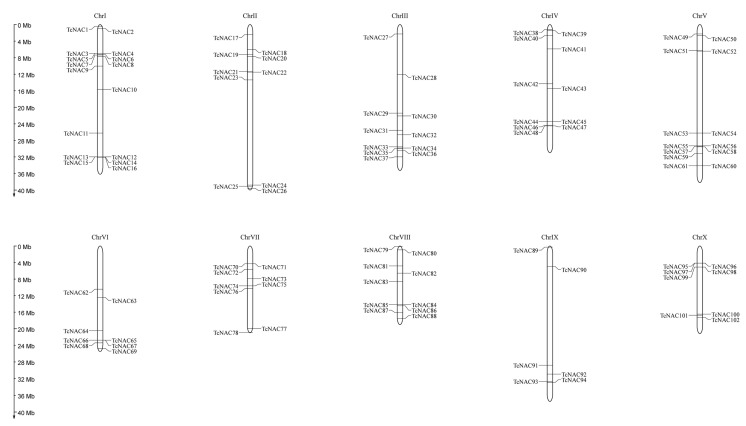
Distribution of *TcNAC* genes among 10 chromosomes. Vertical bars represent the chromosomes of *Theobroma cacao*. The chromosome number is to the top of each chromosome. The scale on the left represents chromosome length.

The distribution of the cacao tree *NAC* genes throughout the genome was analyzed using MG2C. Based on the annotation information of *Theobroma cacao* genome downloaded from Ensembl Plants, 102 cacao tree *NAC* genes were mapped to the chromosomes. The results (Figure 3) showed that 102 cacao *NAC* genes were distributed on 10 chromosomes. There were 16, 10, 11, 11, 13, eight, nine, 10, six, and eight *NAC* genes on chromosomes I–X, respectively. Chromosome I had the most *TcNAC* genes (16, 15.15%), while chromosome IX had only six *TcNAC* genes (~5.8%). Chromosome IX had the fewest *TcNAC* genes. These results indicate an imbalance in the distribution of the cacao tree *NAC* genes among chromosomes, which may be due to differences in chromosome size and structure. On chromosome I, *TcNAC065*, *TcNAC066*, and *TcNAC067* overlap, but these three genes are located far apart in the phylogenetic tree, suggesting that they may have different biological functions. The same phenomenon occurs on chromosome VII (*TcNAC070* and *TcNAC071*, *TcNAC074* and *TcNAC075*), chromosome VIII (*TcNAC084* and *TcNAC085*), and chromosome X (*TcNAC095* and *TcNAC096* and *TcNAC097*, *TcNAC098*, and *TcNAC099*).

Co-linear analysis of the *NAC* family in cacao trees revealed 12 tandem repeat sequences among 102 *TcNAC* genes. The fragment repeat gene was present on chromosomes I, IV, V, VI, VIII, and X, while there were no tandem repeat genes on chromosome II, III, VII, or IX.

According to the KaKs calculator (Table 1), only one of the 12 tandem repeat sequences had Ka/Ks values >1 (*TcNAC047*/*TcNAC046*), which indicates that only one tandem repeat sequence was positively selected for to facilitate adaptive genetic variation; that pair of genes may play an active role in species evolution and could serve as the focus of follow-up studies. All other genes had Ka/Ks values <1, indicating negative selection during evolution, which reduces the rate of change in aa profile. Overall, the results showed that most *NAC* genes are slowly evolving.

## 4. Discussion

The study of transcription factors is currently a major focus in biological research. Transcription factors regulate the expression of downstream genes and are also important for regulating various physiological activities. In recent years, a series of transcription factors regulating drought, high salt, low temperature, hormonal, and pathogenic reactions were isolated from plants. These transcription factors may enhance the resistance and adaptability of plants to various stresses. Gene research can clarify the transmission mechanism of plant stress signals and inform plant resistance breeding. Moreover, genomic research can provide a theoretical basis for gene transformation technology, to obtain highly resistant transgenic organisms.

The cacao tree is an important economic tree species. In 2011, the whole-genome sequence of the cacao tree (*Theobroma cacao*) was completed, which made it possible to analyze the various families of cacao trees using bioinformatics. Data mining and phylogenetic techniques can be applied to analyze the genome of tree species. In this study, the *NAC* gene was identified within the genome of the cacao tree group, and the *NAC* gene was screened using an HMM. In total, 102 *NAC* genes of the cacao tree were finally obtained, which is close to the number of *NAC* genes in *Arabidopsis* (105) and rice (151) [25,26], thereby indicating that most of the genes of the cacao tree *NAC* genes were not eliminated by environmental selection; instead, they showed high conservation during evolution, although they remain to be studied in detail from an evolutionary perspective.

In this study, we analyzed the physicochemical properties (aa, pI, MW, etc.), gene structure (introns, exons), conservative motifs, phylogenetic trees, gene chromosomal locations, and Ka/Ks values of tandem repeats and promoter cis-acting elements of the *NAC* gene family in the cacao tree (102 *TcNAC* genes). The *NAC* gene family was shown to be rich in acidic aa; moreover, members of the same subfamily were found to be similar, suggesting that they may have the same functions. Furthermore, genes with similar evolutionary relationships had similar structures. Phylogenetic tree analysis of the cacao tree and *Arabidopsis* showed that the *NAC* gene family members were unevenly distributed among subfamilies, and the *NAC* gene family members in both cacao and *Arabidopsis* subfamilies. This suggests that the *NAC* gene family existed before the differentiation of the two species, such that the genes in the subfamilies share a common parent and may have similar functions. Since cacao and *Arabidopsis thaliana* were exposed to different environments during evolution, gene differentiation occurred, and the number of *NAC* genes in their subfamilies, thus, differs. Promoter analysis indicated that the cacao tree *NAC* genes play a role in a variety of stress responses, in turn suggesting that the *NAC* gene family may be involved in the growth and development of the cacao tree. Analysis of the function of the *NAC* family in the cacao tree is in its early stages; gene cloning and expression analyses are needed for verification.

Transcription factor families contain many genes that play key roles in plant stress tolerance. To lay the foundation for further studies of the function of *NAC* genes, this study used a bioinformatics method for deep and systematic analysis of the cacao tree *NAC* gene family. The biological function of the cacao tree NAC transcription factor will be the focus of future research. Studying this gene family could provide rich theoretical resources to inform future gene cloning efforts, as well as useful reference data on the regulation, structure, and function of these genes.

## Figures and Tables

**Table 1:** The Ka/Ks values of *Theobroma cacao* tandem repeat sequences.

Tandem Repeat Sequence	Ka	Ks	Ka/Ks
TcNAC048/TcNAC047	0.069652	0.204593	0.34044
TcNAC055/TcNAC057	0.022392	0.046771	0.47876
TcNAC055/TcNAC056	0.031369	0.066627	0.47082
TcNAC056/TcNAC057	0.020842	0.045468	0.45839
TcNAC047/TcNAC046	0.071078	0.056378	1.26074
TcNAC085/TcNAC084	0.057197	0.128216	0.44609
TcNAC085/TcNAC086	0.051726	0.110318	0.46888
TcNAC003/TcNAC004	0.028539	0.129133	0.22101
TcNAC100/TcNAC101	0.014445	0.046379	0.33115
TcNAC084/TcNAC086	0.053422	0.090511	0.59023
TcNAC063/TcNAC005	0.111896	0.215804	0.51851
TcNAC048/TcNAC046	0.077084	0.210968	0.36538

## Supplementary Materials

The following are available online at https://www.mdpi.com/2073-4425/11/1/35/s1: Supplementary File 1: A complete list of *NAC* gene sequences identified in the present study; Supplementary File 2: Features of *NAC* genes identified in *Theobroma cacao*; Supplementary File 3: Sequence logos for the conserved motifs of *Theobroma cacao* NAC domain proteins; Supplementary File 4: Predicted cis-elements in *TcNAC* promoters.
